# Surgical Outcomes in Macular Telangiectasia Type 2-Related Macular Holes: A Report on Four Patients

**DOI:** 10.1155/2020/8884638

**Published:** 2020-12-07

**Authors:** Kinza T. Ahmad, Joseph Chacko, Ahmed B. Sallam, David B. Warner, Sami H. Uwaydat

**Affiliations:** Jones Eye Institute, University of Arkansas for Medical Sciences, 4301 W. Markham St. Little Rock, AR 72205, USA

## Abstract

**Introduction:**

The few published studies that exist on the surgical outcomes of full-thickness macular hole (FTMH) repair in Macular Telangiectasia (MacTel) Type 2 report poor rates of hole closure of around 30%. This study is the largest case series of patients with FTMH in MacTel Type 2 and describes an 80% hole closure rate.

**Purpose:**

/

**Aim:**

To describe the outcomes of four patients who underwent surgery for FTMH associated with MacTel Type 2.

**Methods:**

A retrospective review of clinical, surgical, and imaging data of five eyes in four patients with MacTel Type 2 FTMH who underwent pars plana vitrectomy (PPV) with internal limiting membrane (ILM) peeling and 30% SF_6_ or 15% C_3_F_8_ gas tamponade within 3-9 months of initial vision decline.

**Results:**

Visual acuity (VA) at the time of surgery ranged from 20/50 to 20/200. Successful hole closure was achieved in four out of five eyes, and final VA ranged from 20/20 to 20/40 at follow-up visits greater than 20 months postoperatively. The single eye that did not achieve hole closure had a final VA of 20/60.

**Conclusion:**

Our case series describes higher hole closure rates and better final VA than previously published reports for macular hole surgery in patients with MacTel Type 2 FTMH.

## 1. Introduction

Macular Telangiectasia (MacTel) Type 2 is an idiopathic bilateral foveal degeneration characterized by neurosensory atrophy and changes in the macular capillary network [1]. Clinical findings include angiographic perifoveal vascular leakage, telangiectatic vessels more prominent in the temporal macula, and cystic foveal changes. Full-thickness macular holes (FTMH) in MacTel Type 2 are rare and can be seen at any stage [2]. Given the rarity of FTMH in MacTel Type 2, few published studies exist on the surgical outcomes of these patients. Existing studies have reported that pars plana vitrectomy (PPV) in this setting has a guarded prognosis with a closure rate of about 25-30% [2–7]. In this series, we report successful surgical outcomes of PPV for FTMH in 5 eyes (4 patients) with angiographically confirmed MacTel Type 2. We also report on the postoperative angiographic findings that show persistent vascular leakage following hole closure.

## 2. Methods

We conducted a retrospective review of the clinical, surgical, and imaging data of 5 eyes in 4 patients who underwent surgery for FTMH associated with MacTel Type 2 at the University of Arkansas for Medical Sciences Hospital in Little Rock, AR. Each eye underwent PPV with indocyanine green- (ICG-) assisted internal limiting membrane (ILM) peeling, gas tamponade, and one week of strict prone positioning. All vitrectomies were performed by one surgeon (SHU). Eyes were evaluated at various time points with fundus photographs, fluorescein angiography (FA), optical coherence tomography (OCT), and OCT-angiography (OCT-A) imaging.

## 3. Case Presentations

The characteristics, surgical methods, and outcomes of the cases are summarized in Table 1.

### 3.1. Case 1

**Figure 1:**
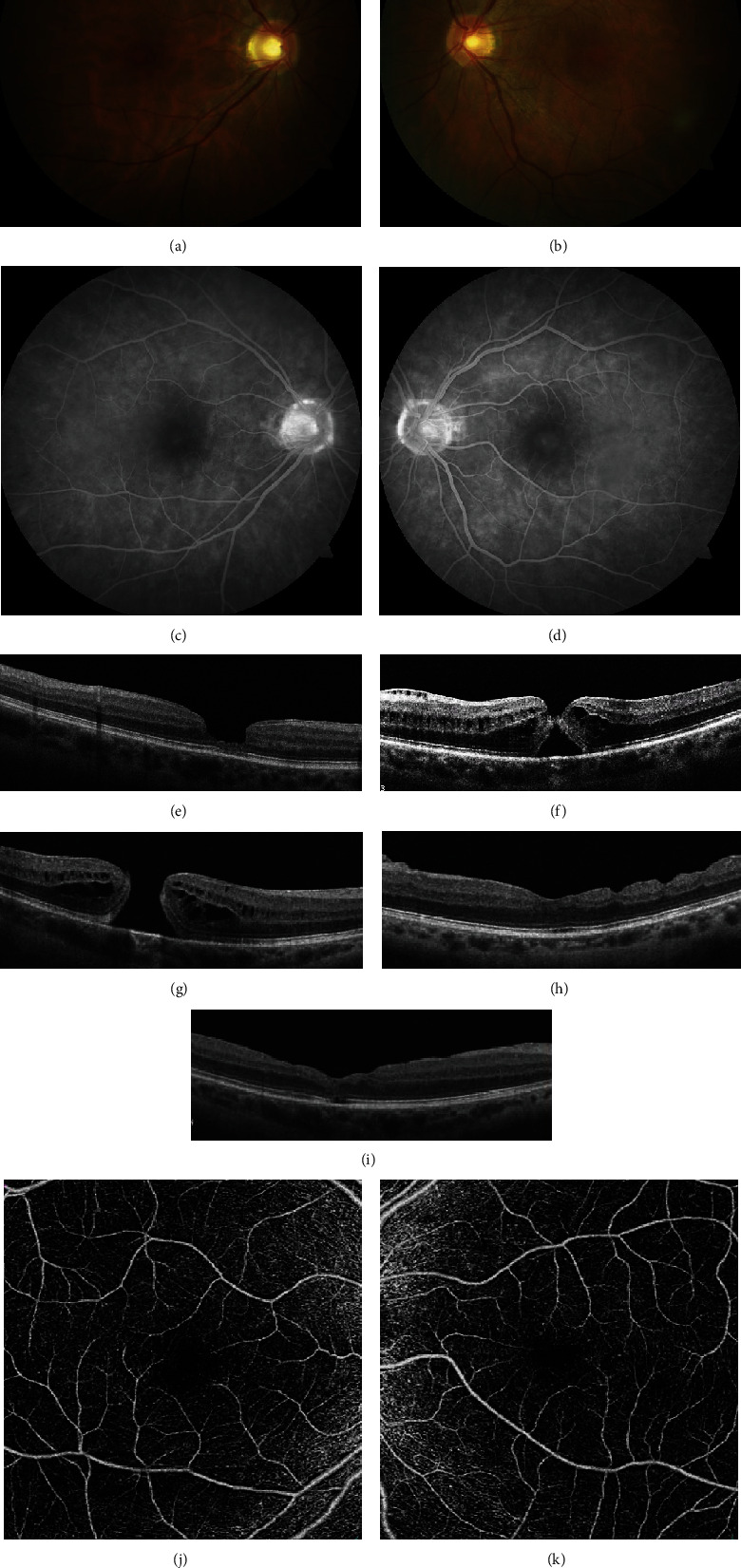
Case 1. Fundus image (a, b) and FA (c, d) show features consistent with MacTel Type 2 in both eyes. Preoperative OCT at initial presentation shows foveal thinning with outer neurosensory atrophy OD (e) and a full-thickness macular hole (FTMH) OS (f). The right eye developed a FTMH at a later visit (g). Later, postoperative OCT of each eye shows complete hole closure in each eye (h, i). Postop OCT-A photos in the right eye show areas of capillary dropout in the superficial bed (j) and in the left eye show telangiectasias in the superficial capillary bed temporal to the fovea with areas of capillary dropout (k).

A 62-year-old woman presented with a three-month history of blurry vision in her left eye. On examination, her best corrected visual acuity (BCVA) was 20/30 in the right eye and 20/80 in the left eye. Anterior segment evaluation revealed a 2+ nuclear sclerotic cataract (NSC) in both eyes. On fundus examination, there was a loss of the foveal reflex in the right eye and a FTMH in the left eye (Figures 1(a) and 1(b)). OCT showed significant foveal thinning with outer neurosensory atrophy in the right eye (Figure 1(e)) and a FTMH with an associated epiretinal membrane (ERM) in the left eye (Figure 1(f)). Fluorescein angiography was significant for mild leakage temporal to the fovea in both eyes with a window defect corresponding to the macular hole in the left eye (Figure 1(d)). The OCT and FA findings were consistent with the diagnosis of MacTel Type 2. The patient underwent a 23-gauge PPV with ERM/ILM peeling and injection of 30% SF_6_, combined with phacoemulsification and intraocular lens (IOL) implantation. Strict prone positioning was maintained for 5 days postoperatively. At the 2-week postoperative visit, closure of the macular hole was confirmed by OCT (Figure 1(h)). The hole remained closed at her most recent visit 37 months postop with a BCVA of 20/20.

The patient presented 10 months following surgery on her left eye with a 2-week history of vision loss in the right eye. The vision had dropped to 20/80, and OCT confirmed the presence of a FTMH (Figure 1(g)). She again underwent phacoemulsification with IOL implantation and PPV with ILM peeling and 30% SF_6_ gas tamponade in the right eye approximately 4 weeks after presentation. Postoperative closure of the macular hole was confirmed by OCT 4 days after surgery. The hole remained closed on her most recent follow-up visit 26 months after surgery with the BCVA stable at 20/25 (Figure 1(i)). A postoperative OCT-A photo of the right eye shows areas of capillary dropout in the superficial bed (Figure 1(j)) and in the left eye shows telangiectasias in the superficial capillary bed temporal to the fovea with areas of capillary dropout (Figure 1(k)); these are persistent vascular anomalies associated with MacTel Type 2 that have been described in the literature [8].

### 3.2. Case 2

**Figure 2:**
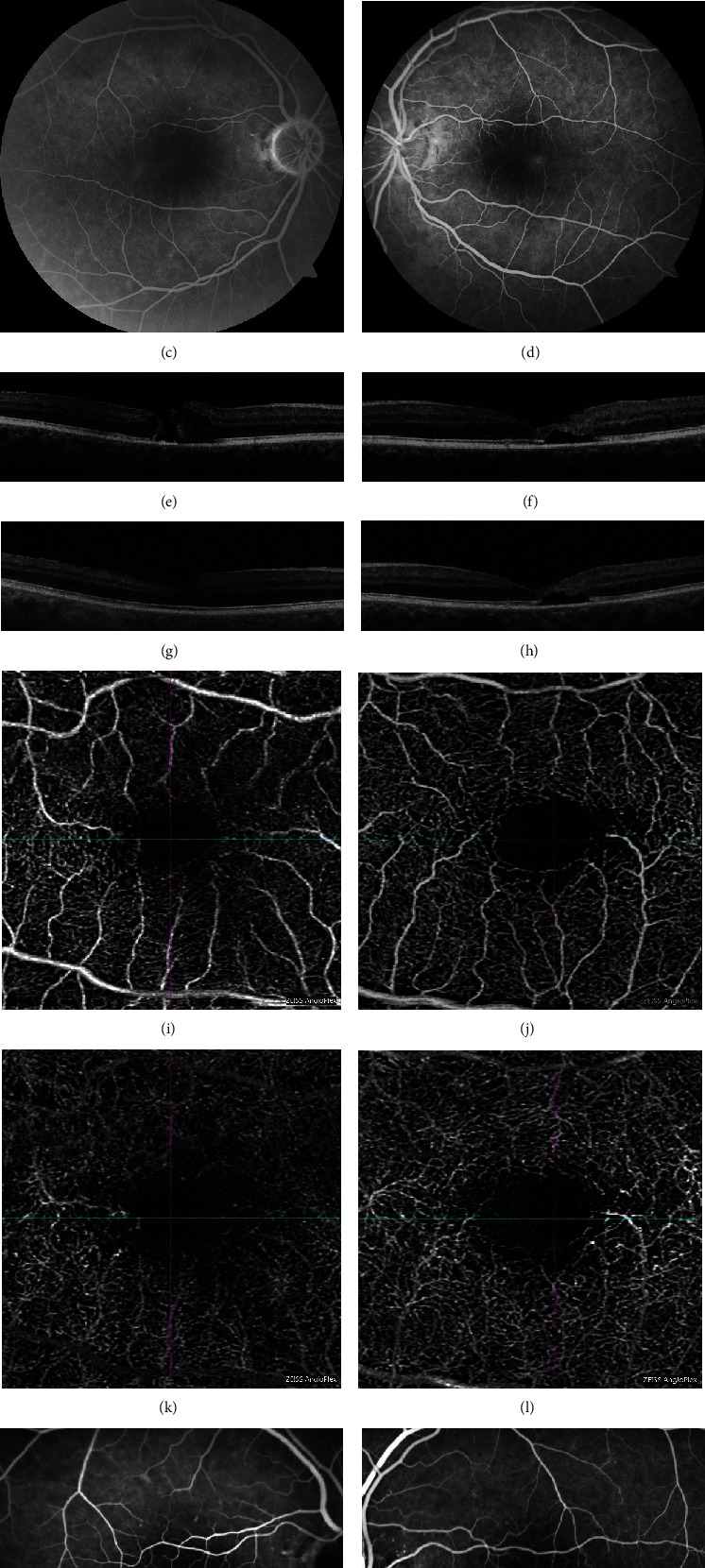
Case 2. Fundus image (a, b) and FA (c, d) show loss of the foveal reflex with hyperfluorescence temporal to the fovea in both eyes. On presentation, OCT shows a FTMH in the right eye with intraretinal cysts and loss of the outer foveal layers in the left eye (e, f). Postoperative OCT of the right eye shows a closed macular hole (g). OCT of the left eye demonstrates a stable lamellar hole (f, h). Postop OCT-A shows right-angled venules and FAZ irregularity as well as significant telangiectasias of the deep vascular network with reduced vascular density (i–l). Postoperative FA shows persistent hyperfluorescence temporal to the fovea (m, n).

A 71-year-old man presented with a few months history of decreased vision in the right eye. BCVA was 20/50 OD and 20/40 OS. Anterior segment examination was significant for 2+ NSC in both eyes. Fundus examination revealed a macular hole in the right eye with pigment clumps temporal to the hole and a lamellar hole in the left macula with intraretinal crystals (Figures 2(a) and 2(b)). OCT confirmed the presence of a full-thickness macular hole in the right eye and a lamellar hole in the left (Figures 2(e) and 2(f)). FA showed leakage temporal to the fovea in both eyes with a window defect in the fovea of the right eye (Figures 2(c) and 2(d)). The diagnosis of MacTel Type 2 was made in both eyes. Four weeks after presentation, a 23-gauge PPV with ILM peeling and fluid-gas exchange with 30% SF_6_ was performed and prone positioning was maintained for 5 days. On the fifth postoperative day, OCT confirmed the closure of the macular hole. Cataract extraction was performed 7 months after PPV at another facility. Twenty-one months after PPV, the macular hole in the right eye remained closed (Figure 2(g)) and BCVA was 20/20-1. In the left eye, the lamellar hole remained stable. Repeat FA revealed significant dye leakage temporal to the fovea in both eyes. Postoperative OCT-A demonstrated remodeling of the deep capillary plexus characteristic of MacTel Type 2 (Figures 2(k) and 2(l)).

### 3.3. Case 3

**Figure 3:**
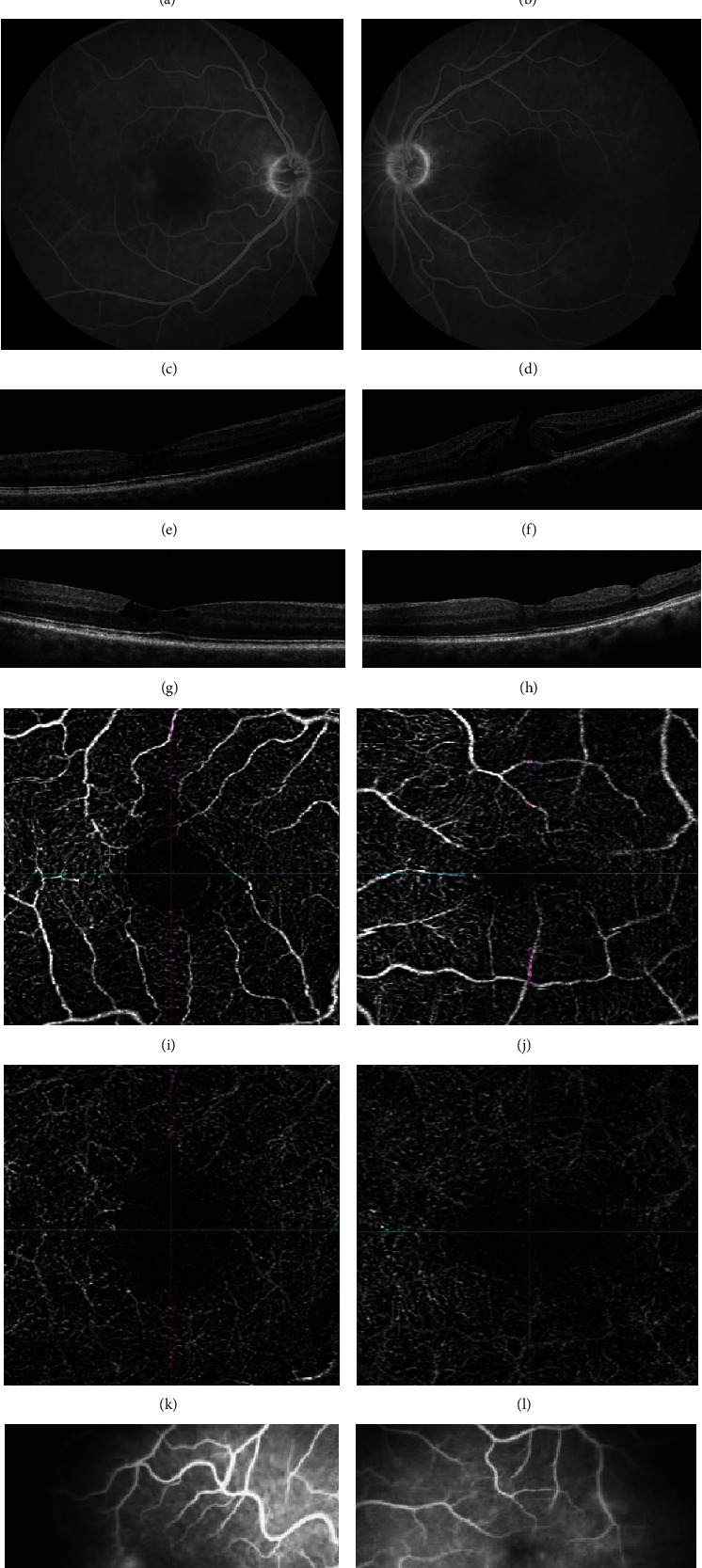
Case 3. Fundus image (a, b) and FA (c, d) show features typical of MacTel Type 2 with leakage temporal to the fovea in both eyes. On presentation, OCT (e, f) shows a FTMH in the left eye and some intraretinal cysts in the right eye. Postoperatively, OCT of the left eye (h) shows a closed macular hole. OCT-A of both eyes (i–l) demonstrates telangiectatic vessels, right-angled venules, and enlarged FAZ, with capillary bed closure temporal to the fovea. Postoperative FA of both eyes shows persistent leakage temporally (m, n).

A 65-year-old lady with a past medical history of hypertension presented with 8 months of decreased vision in the left eye. BCVA was 20/25 in the right eye and 20/200 in the left. Fundus examination revealed a blunted foveal reflex in the right eye and a FTMH in the left eye (Figures 3(a) and 3(b)). FA revealed faint leakage temporal to the fovea in each eye, consistent with the diagnosis of MacTel Type 2 (Figures 3(c) and 3(d)). She underwent 23-gauge PPV with ILM peeling and 30% SF_6_ gas tamponade 7 weeks after presentation. On the 5^th^ postoperative day, the hole was sealed by OCT (Figure 3(g)). The patient underwent cataract extraction in the left eye at another facility. On follow-up, the macular hole in the left eye has remained closed on examination through postoperative month 35 with a BCVA of 20/40. In the right eye, an intraretinal cyst remained stable and vision was maintained at 20/30. Postoperative OCT-A showed persistent features typical of MacTel Type 2 (Figures 3(i)–3(l)). Repeat FA showed significant leakage temporal to the fovea in the right eye and minimal leakage in the left eye (Figure 3(m)).

### 3.4. Case 4

**Figure 4:**
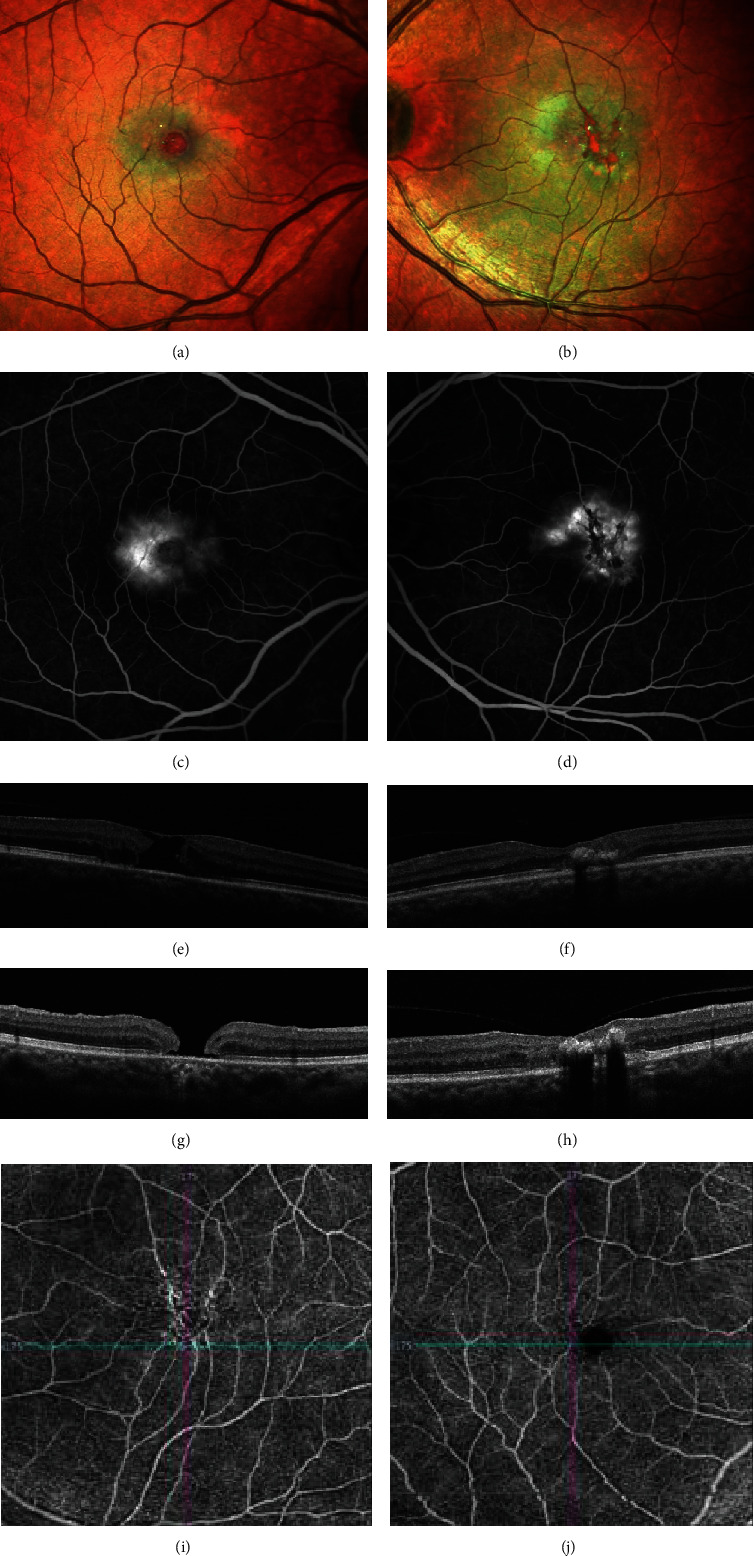
Case 4. Fundus image (a, b) and FA (c, d) show findings consistent with MacTel Type 2. On presentation, OCT confirms a FTMH in the right eye (e). Postoperative OCT (g) shows the hole still being present. OCT-A (i, j) of the superficial layer shows telangiectatic vessels and vascular invasion of the FAZ, especially in the right eye (i).

A 57-year-old diabetic female presented with unknown duration of vision loss in her right eye. BCVA in her right eye at presentation was 20/400 and in the left eye 20/50. On anterior segment examination, she had 2+ NSC in both eyes. Fundus examination of the right eye revealed a blunted foveal reflex while the left eye showed pigmentary changes centrally and temporal scarring of the retinal pigment epithelium, without any evidence of diabetic retinopathy (Figures 4(a) and 4(b)). FA revealed bilateral foveal leakage characteristic of MacTel Type 2 (Figures 4(c) and 4(d)). In the right eye, OCT confirmed the clinically apparent FTMH (Figure 4(e)). In the left eye, OCT demonstrated hyperreflective deposits consistent with the macular scarring that was apparent on fundus examination (Figure 4(f)). Three weeks after presentation, she underwent phacoemulsification with IOL implantation in the right eye. Seventeen days after cataract surgery, her BCVA improved to 20/60 in the right eye. Four weeks after cataract surgery, she had a 23-gauge PPV with ILM peeling and 15% C_3_F_8_ gas tamponade. The hole failed to close as shown on the postoperative OCT (Figure 4(g)), and her BCVA at the 22-month visit was stable at 20/60 in the right eye.

## 4. Discussion

Surgery for FTMH in the setting of MacTel Type 2 has a guarded prognosis. To date, there are only 16 reported cases that have undergone surgical intervention [2–7, 9]. In these 16 cases, 6 achieved successful hole closure, 3 initially closed but then reopened, and 7 failed to close at all (Table 2). Of the 6 that achieved successful anatomical closure, only 5 had improvement in visual acuity.

The difference in pathophysiology of hole formation is one proposed reason for the much lower success rate of macular hole surgery in MacTel Type 2 as compared to idiopathic macular holes. Idiopathic macular holes are thought to develop from retinal cavitation due to vitreomacular traction [10]. By contrast, in MacTel Type 2, macular holes are postulated to occur from retinal cavitation due to Müller cell degeneration [11]. Histologic analysis also supports this theory as patients with MacTel Type 2 have had reduced expression of Müller cell markers in corresponding areas of the macula [12]. It is thought that the tissue defect from Müller cell atrophy in MacTel Type 2 causes foveal structural instability and leaves an insufficient amount of tissue for the hole to seal. This may account for the historically poor surgical outcomes in MacTel Type 2 FTMH repair. This pathologic process is thought to be independent of the vascular abnormalities in MacTel Type 2. In his initial paper, Gass had proposed a primary role for abnormal retinal vasculature leading to retinal atrophy [13]. However, he later revised this theory to suggest a primary role of parfoveolar Müller cell abnormality since telangiectatic vessels often did not develop till later in the disease [14].

Rishi and Kothari first reported on the surgical management of patients with FTMH in MacTel Type 2. In their series of 2 patients, one achieved successful hole closure and improvement in vision from 20/100 to 20/63, while the other case exhibited initial closure followed by reopening at 4 months and no functional vision improvement [4]. Several case series have followed since then, all with overall poor closure rates. Issa et al. described 2 patients with MacTel Type 2 in whom the FTMH did not close [2]. In 2010, Gregori and Flynn reported on 2 patients both of whom had initial hole closure with reopening of the hole in one of the patients after 4 months [3]. In the patient with successful hole closure, VA improved from 20/50 to 20/30. In 2011, Shukla described successful hole closure in 1 patient with VA improvement from 20/80 to about 20/30 at the 11-month postoperative visit [5]. This was followed by Karth et al. who described FTMH in 4 patients with stage 3 and 4 MacTel Type 2 [6]. The holes successfully closed in one patient, reopened in one, and failed to close in two. In the single patient with successful closure, VA improved from 20/200 to 20/30. This study was followed by Patel and Flaxel who reported on 2 patients in whom the hole did not close following attempted surgical repair [7]. Most recently, a group from Korea published the first case series on Asian patients [9]. They reported on 4 patients with MacTel Type 2 and FTMH with an average thickness of 345 *μ*m. Three of the 4 patients had successful hole closure, and the fourth patient had initial closure with recurrence after 1 month. Among the 3 patients with successful closure, 2 had improved vision—from 20/125 to 20/32 at postop month 43 in one patient and from 20/200 to 20/32 at postop month 6 in the second patient. The third patient with successful closure had worsening of vision from 20/200 to 20/400 at 10 months postoperatively. The results of all these described studies are summarized in Table 2. Before the addition of our cases, the successful hole closure rate was 37.75% with the inclusion of all the most recent cases. By contrast, the rate of hole closure in our cases alone was 80%. The inclusion of our 5 cases to the previously existing data increased the hole closure rate to 47.6%.

Our series of surgical outcomes in 5 eyes with FTMH in MacTel Type 2 is the largest to date and has the highest success rate of hole closure among currently published studies. As described above, we had successful hole closure in 4 out of 5 eyes with final visual acuities ranging from 20/20 to 20/40 at the follow-up visits at least 21 months out from surgery in each patient. There are several possibilities to explain the higher success in our series compared to previously reported cases. We performed ILM peeling in all eyes from major vascular arcade to arcade. Only one of the previously published studies detailed the extent of ILM peeling, and this was the one by Lee et al. Interestingly, similar to us, this group performed ILM peeling of the major vascular arcades as well and three out of four holes remained closed in their series. Given the higher success rate in patients that undergo a larger extent of ILM peeling, it may be that this is what leads to better results in FTMH repair in MacTel Type 2. ILM peeling may activate Müller cells, stimulating the secretion of collagen, basement membrane components, and inflammatory factors which may subsequently activate glial cell-mediated closure of macular holes as suggested in histological studies [15]. Despite the depletion of Müller cells, the foveal structure may be reinforced by the remaining Müller cells, contributing to the hole closure. Another possibility that may have contributed to our successful surgical outcomes was our reinforcement of strict head positioning for 5 days postoperatively. It may also have helped that our patients underwent surgery at an earlier stage of MacTel, as most of our patients did not have the foveal intraretinal pigment changes observed in the later stages of MacTel Type 2. Of note, FA and OCT-A in three of our patients with successful hole closure revealed persistence of foveal vascular anomalies after surgery, evidenced by fluorescein leakage on FA and the presence of abnormal capillary beds on OCT-A. This supports the hypothesis that vascular incompetence is unlikely to have contributed to macular hole formation [14] as 4 of the 5 macular holes remained closed more than 21 months after surgery.

Our study is limited by the small number of cases; however, this is expected given the rarity of the disease. Additionally, there is a possibility that the visual improvement seen in our first patient may have been from the phacoemulsification component of the surgery. However, although this patient had combined cataract and retinal surgery, anatomic closure of the hole was still achieved and it is likely that both surgeries contributed to the final improvement in VA. Additionally, the second and third patients did not have concomitant cataract surgery and these patients also had improvements in VA in addition to anatomical closure. Strengths of our case series include including OCT-A imaging in all five eyes and long-term follow-up of greater than 20 months. Additionally, given that the same vitreoretinal surgeon performed all surgeries, the surgical technique employed was uniform among all five eyes.

## 5. Conclusion

In conclusion, our case series suggests that a higher rate of success may be achievable in surgical repair of FTMH in MacTel Type 2. Specifically, characteristics that may have contributed to a greater chance of success include operating at an earlier stage of MacTel, operating promptly after initial symptom onset, a more thorough ILM peeling, and strict prone positioning postoperatively. Our data may help inform the preoperative discussion surgeons have with their patients. Finally, our findings of persistent leakage on angiography and lack of vascular remodeling on OCT-A despite successful hole closure suggest that vascular leakage in MacTel Type 2 is unlikely to contribute to the pathophysiology of hole formation or closure.

## Figures and Tables

**Table 1:** Summary table of patient characteristics, surgical methods, and outcomes of patients with FTMH associated with MacTel Type 2.

	Case 1 OD	Case 1 OS	Case 2 OD	Case 3 OS	Case 4 OD
Age (years)	62	62	71	65	57
Gender	Female	Female	Male	Female	Female
Preoperative BCVA	20/80	20/80	20/50	20/200	20/60
Time from symptom onset to surgery	1 month	3 months	4 months	9 months	Unknown
Macular hole size (*μ*m)					
Diameter	410	246	225	184	493
Maximum height	426	424	340	403	309
Posterior vitreous detachment	Yes	Yes	No	No	No
Surgical procedure	PPV, ICG-assisted ILM peeling, cataract surgery done at the time of PPV	PPV, ICG-assisted ILM peeling, cataract surgery done at the time of PPV	PPV, ICG-assisted ILM peeling	PPV, ICG-assisted ILM peeling	PPV, ICG-assisted ILM peeling, cataract surgery done 1 month prior to PPV
Tamponading agent	30% SF_6_	30% SF_6_	30% SF_6_	30% SF_6_	15% C_3_F_8_
Positioning, duration	Prone, 1 week	Prone, 1 week	Prone, 1 week	Prone, 1 week	Prone, 1 week
Anatomical hole closure	Closed hole	Closed hole	Closed hole	Closed hole	Not closed
EZ and ELM integrity on most recent OCT	Yes	Yes	Yes	Yes	No
BCVA at last follow-up	20/25	20/20	20/20-1	20/40-1	20/60
Length of follow-up	26 months	37 months	21 months	35 months	22 months

PPV: pars plana vitrectomy; ILM: internal limiting membrane; ICG: indocyanine green; EZ: ellipsoid zone; ELM: external limiting membrane; BCVA: best corrected visual acuity; SF_6_: sulfur hexafluoride; C_3_F_8_: perfluoropropane.

**Table 2:** Table of anatomical results from published cases of patients with idiopathic Macular Telangiectasia Type 2 and FTMH undergoing surgery [2–7, 9].

Year	Author	Number of eyes	Hole closed	Hole reopened	Hole not closed
2008	Rishi and Kothari	1	0	0	1
2009	Issa et al.	2	0	0	2
2010	Gregori and Flynn	2	1	1	0
2011	Shukla	1	1	0	0
2014	Karth et al.	4	1	1	2
2015	Patel et al.	2	0	0	2
2019	Lee et al.	4	3	1	0
	Subtotal	16	6 (37.5%)	3 (18.75%)	7 (43.75%)
Our study		5	4 (80%)	0 (0%)	1 (20%)
	Total	21	10 (47.6%)	3 (14.2%)	8 (38.1%)

## Abbreviations

FTMH: Full-thickness macular hole
MacTel: Macular Telangiectasia
PPV: Pars plana vitrectomy
ILM: Internal limiting membrane
SF_6_: Sulfur hexafluoride
C_3_F_8_: Octafluoropropane
VA: Visual acuity
ICG: Indocyanine green
FA: Fluorescein angiography
OCT: Optical coherence tomography
OCT-A: Optical coherence tomography-angiography
BCVA: Best corrected visual acuity
NSC: Nuclear sclerotic cataract
ERM: Epiretinal membrane
IOL: Intraocular lens.

## References

[1] Green W. R., Quigley H. A., de la Cruz Z., Cohen B. (1980). Parafoveal retinal telangiectasis. Light and electron microscopy studies. *Transactions of the Ophthalmological Societies of the United Kingdom*.

[2] Charbel Issa P., Scholl H. P. N., Gaudric A. (2009). Macular full-thickness and lamellar holes in association with type 2 idiopathic macular telangiectasia. *Eye*.

[3] Gregori N., Flynn H. W. (2010). Surgery for full-thickness macular hole in patients with idiopathic macular telangiectasia type 2. *Ophthalmic surgery, lasers and imaging retina.*.

[4] Rishi P., Kothari A. R. (2008). Parafoveal telangiectasia (PFT) has been associated with changes in macular architecture and macular holes (lamellar and full thickness). *Retina*.

[5] Shukla D. (2011). Evolution and management of macular hole secondary to type 2 idiopathic macular telangiectasia. *Eye*.

[6] Karth P. A., Raja S. C., Brown D. M., Kim J. E. (2014). Outcomes of macular hole surgeries for macular telangiectasia type 2. *Retina*.

[7] Patel P., Flaxel C. (2015). Visual and anatomic outcomes in eyes with idiopathic juxtafoveal macular telangiectasia (MacTel) and full thickness macular holes undergoing surgical repair. *Enliven: Clin Ophthalmol Res*.

[8] Nalcı H., Şermet F., Demirel S., Özmert E. (2017). Optic coherence angiography findings in type-2 macular telangiectasia. *Turkish journal of ophthalmology*.

[9] Lee S. C., Hwang D. J., Lee K. M., Park Y. S., Sohn J. H. (2019). Surgical outcomes of macular telangiectasia type 2 associated with macular hole. *Journal of Retina*.

[10] Spaide R. F. (2000). Closure of an outer lamellar macular hole by vitrectomy: hypothesis for one mechanism of macular hole formation. *Retina*.

[11] Koizumi H., Slakter J. S., Spaide R. F. (2007). Full-thickness macular hole formation in idiopathic parafoveal telangiectasis. *Retina*.

[12] Powner M. B., Gillies M. C., Zhu M., Vevis K., Hunyor A. P., Fruttiger M. (2013). Loss of Müller's cells and photoreceptors in macular telangiectasia type 2. *Ophthalmology*.

[13] Gass J. D., Oyakawa R. T. (1982). Idiopathic juxtafoveolar retinal telangiectasis. *Archives of Ophthalmology*.

[14] Gass J. D. M. (2000). Histopathologic study of presumed parafoveal telangiectasis. *Retina*.

[15] Hisatomi T., Tachibana T., Notomi S. (2017). Incomplete repair of retinal structure after vitrectomy with internal limiting membrane peeling. *Retina*.

